# Weekly paclitaxel plus trastuzumab in metastatic breast cancer pretreated with anthracyclines-a phase II multipractice study

**DOI:** 10.1186/1471-2407-12-165

**Published:** 2012-05-04

**Authors:** Matthias John, Axel Hinke, Martina Stauch, Heiner Wolf, Benno Mohr, Hans-Joachim Hindenburg, Jens Papke, Joachim Schlosser

**Affiliations:** 1 Practice for Gynecology, Dr.-Doerffel-Strasse 1, 08371, Glauchau, Germany; 2 WiSP Research Institute, Karl-Benz-Str. 1, 40764, Langenfeld, Germany; 3 Practice for Medical Oncology, Niederbonner Arnoldstrasse 2, 96317, Kronach, Germany; 4 Practice for Medical Oncology, Arnoldstrasse 18, 01307, Dresden, Germany; 5 Practice for Medical Oncology, Breite Strasse 52, 13597, Berlin, Germany; 6 Practice for Medical Oncology, Pichelsdorfer Str. 105, 13595, Berlin, Germany; 7 Praxis for Medical Oncology, Rosa-Luxemburg-Strasse 6, 01844, Neustadt/Sachsen, Germany; 8 Practice for Gynecology, Clausstrasse 76-80, 09126, Chemnitz, Germany

## Abstract

**Background:**

The 3-weekly combination of trastuzumab and paclitaxel has been approved for the treatment of advanced breast cancer based on a large pivotal study. However, mono and combination chemotherapy trials suggest that weekly paclitaxel has a better therapeutic index, especially in the palliative setting. The present trial examined the efficacy and safety of weekly paclitaxel over a limited duration combined with continued trastuzumab in HER2+ patients.

**Methods:**

Patients with histologically confirmed metastatic breast cancer overexpressing HER2 were eligible if pretreated with anthracycline in either the adjuvant or palliative setting. Treatment consisted of weekly trastuzumab (2 mg/kg/week for up to one year after a loading dose of 4 mg/kg in week 1) and paclitaxel (90 mg/m², administered in weeks 1–6 and 8–13).

**Results:**

Twenty-seven German centers enrolled 121 patients. The median number of metastatic sites was two (range 1–5); 38% of patients had received chemotherapy for advanced disease. After a median 42 weeks of trastuzumab treatment, limited by disease progression in roughly half the patients, a best objective response rate (complete response + partial response) of 76% was achieved, including complete remissions in 29%. 74% of patients lived without tumor progression at six months. Median progression-free and overall survival were 9.4 (95% confidence interval [CI]: 8.1–11.3) and 22 months (95% CI: 17–46). After alopecia, Common Toxicity Criteria grade ≥2 toxicity was predominantly hematological (leukopenia [31%] and anemia [41%]); however, thrombocytopenia occurred in only 5%. Neurotoxicity was remarkably low. Two cardiac events (grades 2 and 3) were presumed treatment-related.

**Conclusions:**

Weekly paclitaxel plus trastuzumab allows an increased dose density and offers an attractive and effective alternative to the conventional schedule. Limiting the duration of cytotoxic therapy to 3 months seems to be an option to reduce neurotoxicity without impairing long-term outcome.

## Background

Recent advances in understanding cancer biology have spurred the development of a plethora of targeted agents that have revolutionized the treatment of a number of malignancies. Their mechanism of action often depends on specific molecular features allowing for individualized strategies, in contrast to the non-selective approaches of the conventional chemotherapy era. The best known and most successful example was the discovery that 20 to 25% of advanced breast cancer patients overexpress human epidermal growth factor receptor type 2 (HER2) [1]. This led to the development of trastuzumab (Herceptin®), a humanized monoclonal antibody against this epitope [2] that shows considerable antitumor efficacy as a single agent even in heavily pretreated patients with advanced disease [3,4].

Nevertheless, based on preclinical evidence of synergy [5], the major breakthrough of this targeted approach was to combine the antibody with cytostatic drugs, in both early and advanced disease [2]. The pivotal randomized phase III study on combination trastuzumab in advanced breast cancer allowed for two chemotherapy backbones: either doxorubicin/cyclophosphamide or single-agent paclitaxel, both given 3-weekly [6]. While added trastuzumab increased response and survival rates in each of these strata, combination with anthracyclines unexpectedly increased cardiotoxicity [7] (although less markedly with epirubicin [8]), impeding its widespread use and favoring taxane-oriented strategies.

During the same period evidence emerged that weekly (rather than 3-weekly) paclitaxel achieved high antitumor activity thanks to a considerably increased dose intensity and/or densitiy, yet with no apparent increase in overall toxicity [9,10]. We therefore assessed the efficacy and safety of weekly paclitaxel over a recommended duration of just 3 months while continuing trastuzumab in HER2-positive patients with advanced breast cancer previously treated with anthracyclines.

## Methods

The non-commercial multicenter study was conducted according to the principles of the Declaration of Helsinki (1996 version). Approval was gained from the institutional review boards of all 27 participating centers and each patient gave prior written informed consent.

### Eligibility criteria

Patients with histologically confirmed metastatic breast cancer overexpressing HER2 were eligible if pretreated with anthracycline in either the adjuvant or palliative setting. HER2 positivity was defined as 2+ or 3+ overexpression using the DAKO HercepTest™, confirmed by fluorescence in-situ hybridization (FISH) if 2+. Non-inclusion criteria were >1 chemotherapy for advanced disease, taxane or trastuzumab pretreatment, brain metastases, Eastern Cooperative Oncology Group (ECOG) performance status >1 at screening, pregnancy or lactation, childbearing potential without reliable contraception, clinically significant cardiac disease, neutrophils <1500/μl, platelets <75,000/μl, and total bilirubin and creatinine >1.5 × the upper limit of normal.

### Study treatment

Patients received an intravenous (iv) loading dose of trastuzumab 4 mg/kg body weight on day 0 over an infusion duration of 90 min, followed by an infusion of paclitaxel 90 mg/m^2^ over 1 hour on day 1. From day 8, the same paclitaxel regimen and infusions of trastuzumab 2 mg/kg body weight were administered on the same day every week. The cytotoxic drug was given over two 6-week blocks separated by a 14-day break, with optional prolongation at the physician’s discretion; the antibody was continued weekly until disease progression. Premedication (all iv) was dexamethasone 10 mg, clemastine 2 mg, and ranitidine 50 mg.

The paclitaxel dose was reduced to 75 mg/m^2^ in the event of grade 3/4 hematotoxicity, and stopped completely in the event of grade 3 neurotoxicity or severe hypersensitivity reactions. Termination of trastuzumab was considered in the event of persisting asymptomatic deterioration of left ventricular ejection fraction or symptomatic heart disease.

### Evaluation of safety and efficacy

Baseline documentation included demographics, medical and treatment history, cardiac function, and HER/2neu status. Vital signs, ECOG performance status, and complete blood counts were recorded at baseline and each weekly visit, serum chemistry at baseline and 4-weekly. Tumor assessment comprised physical examination and radiology at baseline, after each paclitaxel treatment block, and 8-weekly thereafter. Tumor response and progression were evaluated using World Health Organization (WHO) criteria. Time to progression was defined as the period between study enrolment and progression or death, whichever occurred first. Cotreatments and adverse events (graded using National Cancer Institute Common Toxicity Criteria NCI-CTC) were recorded continuously throughout the study. Electrocardiography and echocardiography were repeated 12-weekly.

### Statistical aspects

The primary objective of this phase II study was the progression-free survival (PFS) rate at 6 months. 115 patients were required to discriminate a promising 50% PFS rate from a futile finding of 35% with both type I and II errors of 5% [11]. All other parameters were analyzed descriptively in terms of standard distribution parameters such as rates, means with standard deviations, quartiles, and ranges. Event-related data (PFS, overall survival) were estimated using the product limit method of Kaplan and Meier [12]. Two-sided logrank tests were used to compare prognostic subgroups [13].

## Results

Twenty-seven German centers in the FAKT Study Group enrolled 121 patients from February 2001 to July 2004. Ten proved ineligible: eight had not received anthracycline pretreatment, one had been pretreated with paclitaxel, and one was found to have been enrolled retrospectively. A further three patients were removed from the analysis due to missing documentation or serious protocol violation (radiotherapy to the only site of disease), leaving 108 cases for the baseline and safety analyses. Between one and three patients had insufficient data for the analysis of the respective efficacy parameters. Disease was aggressive in most patients, with a median of two organ sites involved and visceral lesions present in about 80% (Table 1). Most patients had received an anthracycline in the adjuvant setting, with the result that a high proportion of patients (63%) received their first-line palliative chemotherapy in this study. Thirteen patients (12%) had a history of heart disease.

**Table 1:** Patient and tumor characteristics (n = 108)

	**n/estimation**	**%**
**Age (years)**		
Mean ± standard deviation	58.4 ± 10.2	
Median (range)	59 (29–78)	
**ECOG performance status on chemotherapy day 1**		
0	42	39
1	57	53
2	8	7
**HER2 overexpression**		
2+/FISH +	8	7
3+	97	90
FISH +/no immunohistochemistry or other	3	3
**Tumor grade (n = 102)**		
G1	2	2
G2	47	46
G3	53	52
**Metastatic sites at onset of study treatment (n)**		
Mean ± standard deviation	1.8 ± 0.9	
Median, range	2 (1–5)	
**Organ site involvement**		
Local/skin	14	13
Lymph nodes, non-regional	20	19
Bone	41	38
Liver	48	44
Lung	49	45
Pleural effusion	13	12
Genitourinary	1	1
Other	9	8
**Previous treatment**		
Neoadjuvant chemotherapy	11	10
Adjuvant chemotherapy	74	69
Adjuvant endocrine therapy	45	42
Palliative chemotherapy	40	37
Palliative endocrine therapy	34	31

Abbreviations

ECOG: Eastern Cooperative Oncology Group.

FISH: fluorescence in-situ hybridization.

### Efficacy

**Figure 1:**
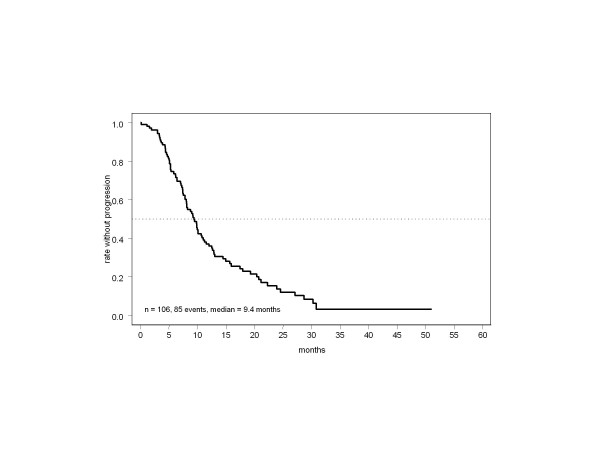
Time to progression in the total population.

**Figure 2:**
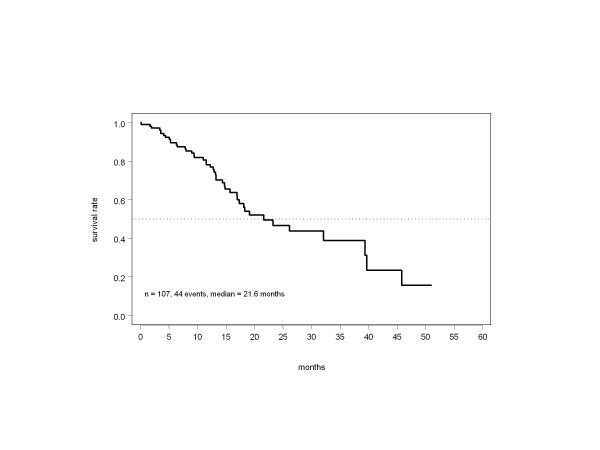
Overall survival in the total population.

The recommended duration of paclitaxel treatment was followed in most patients. Cycle numbers were 12 (median) and 12.6 (mean); 16% of patients received fewer than 12 cycles due to early progression or toxicity; 73% and 12% received 12 and >12 cycles. The median number of trastuzumab doses was 42; antibody therapy lasted ≥1 year in almost half the patients (48%). Treatment withdrawal due to severe toxicity (6%) or patient preference (12%) was uncommon. Tumor response was assessable in 105 patients. The overall objective remission rate was 76% (95% confidence interval [CI]: 67–84%), with 30 (29%) complete responses (CR) and 50 (48%) partial responses (PR), equivalent to a response rate of 74% based on an intention-to-treat population of all eligible cases. Only 7% of patients had direct progression with no period of at least stable disease while on study treatment. Based on a follow-up period of up to more than 4 years and 85 observed events, median PFS was 9.4 months in the total population (Figure 1), with a 6-month PFS rate of 74% (95% CI: 65–83%), representing a highly promising result with respect to the predefined primary endpoint. Almost half the patients were alive more than 2 years after entering the study (Figure 2).

### Prognostic factors

**Figure 3:**
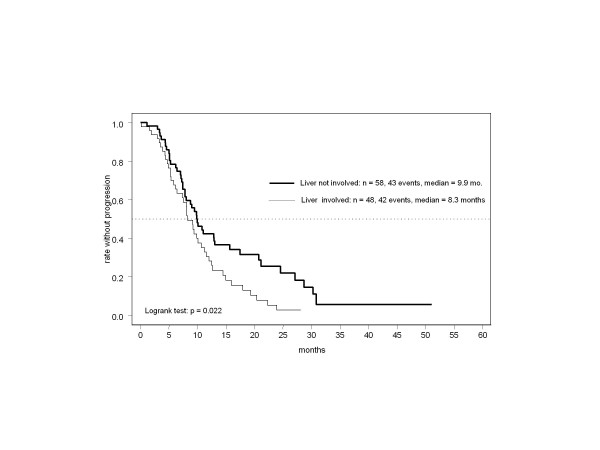
Time to progression by hepatic involvement.

**Figure 4:**
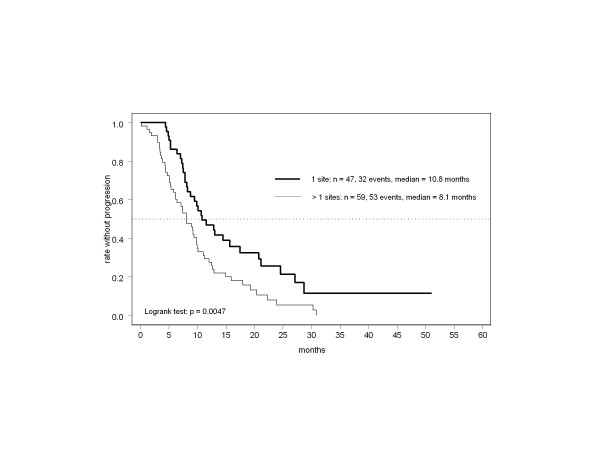
Time to progression by number of metastatic sites.

The impact of several prognostic characteristics on the primary endpoint was analyzed. PFS was significantly longer in the absence of hepatic involvement (*P* = 0.022), especially in the subgroup with a generally better prognosis (Figure 3), as in those with only one site of metastasis (Figure 4, *P* = 0.0047). In contrast, neither an ECOG score ≥1 nor palliative pretreatment with chemotherapy were significant predictors of poorer outcome. Hepatic and visceral involvement had no significant impact on tumor remission, but response was significantly more frequent in patients with only one site of disease (91% CR/PR, *P* < 0.0001). CR was most frequently observed in patients with intact performance status and no liver metastases.

### Safety

Leukopenia occurred in most cases, but reached NCI-CTC grade 3/4 in only 14% (Table 2). Mild to moderate anemia was very common, and thrombocytopenia comparatively rare. Peripheral sensory neuropathy and pain were the most frequent severe non-hematological events, typically accompanied by arthralgia and myalgia. There was no instance of grade ≥2 gastrointestinal toxicity, except one case of life-threatening diarrhea. Clinical chemistry showed no grade 3/4 nephrotoxicity; severe liver enzyme elevation occurred in 5%. There were five cases of hypersensitivity, three out of these of severe grade.. Lung function deteriorated severely in five patients, including one case of grade 3 dyspnea, described as cardiotoxicity, but observed in a patient with pleural effusion. Cardiac symptoms (grade 2) were reported in only one patient.

**Table 2:** Adverse event frequency and severity (maximum National Cancer Institute Common Toxicity Criteria [NCI CTC] grade per category)

**Adverse event/ organ system**	**Patients per NCI CTC grade [n (%)]**			
	**grade 1**	**grade 2**	**grade 3**	**grade 4**
**Hematological**				
White blood cells	33 (31)	22 (20)	9 (8)	6 (6)
Granulocytes	27 (26)	16 (15)	5 (5)	3 (3)
Platelets	16 (15)	2 (2)	3 (3)	1 (1)
Hemoglobin	43 (40)	40 (37)	3 (3)	3 (3)
Fever	20 (19)	8 (7)		
**Non-hematological**				
Hair loss	23 (21)	79 (73)		
Nausea	53 (49)	15 (14)		
Vomiting	32 (30)	10 (9)		
Diarrhea	21 (19)	8 (7)		1 (1)
Constipation	18 (17)	1 (1)		
Neurology/Motor	19 (18)	6 (6)	4 (4)	
Neurology/Sensory	28 (26)	16 (15)	5 (5)	
Arthralgia	27 (25)	9 (8)	2 (2)	
Myalgia	20 (19)	8 (7)	3 (3)	
Lung function	11 (10)	8 (7)	2 (2)	3 (3)
Pain	21 (19)	28 (26)	8 (7)	

## Discussion and conclusions

As the first therapeutic antibody approved for solid tumors, trastuzumab was introduced into the first-line treatment of advanced breast cancer based on a pivotal randomized study assessing its efficacy in combination with cytostatic drugs: combination with 3-weekly paclitaxel clearly outperformed chemotherapy alone, achieving an overall response rate of 41%, a median time to progression of 6.9 months, and overall survival of 22 months [6]. This was confirmed by corresponding results of 36%, 7.1 and 32 months in the control arm of a US Oncology trial comparing this combination to a three-drug regimen comprising paclitaxel, trastuzumab, and carboplatin [14]. Smaller phase II studies on the combination of the HER2 antibody with docetaxel have reported response rates of 50 to 70% [15-17].

Weekly paclitaxel proved a dose-dense alternative to the 3-weekly regimen, achieving at least similar activity in breast and lung cancer, and showing some advantages in terms of feasibility and decreased toxicity. The results of our trial, notably a median PFS of 9.4 months and an overall survival of 22 months, compare favorably to the phase III findings mentioned above, despite including 37% of patients with previous palliative chemotherapy. The intention-to-treat response rate of 74% is considerably higher, but this may be due to the more rigorous focus on remission confirmation in pivotal studies. A number of smaller phase II studies have also investigated the weekly administration of both drugs, likewise reporting high response rates of 56 to 74% in patient populations with varying amounts of pretreatment [18-21]. Seidman et al. [20] administered a regimen similar to ours to patients with a comparable rate of palliative pretreatment (30%), and achieved responses in about 70% of HER2-positive patients, with a median response duration of 7 months. The smaller studies by Fountzilas et al. [18] in 34 patients with no cytotoxic pretreatment for advanced disease, and Gori et al. [21] in 25 patients, most of whom had been heavily pretreated, showed similar efficacy with remission rates of 62% and 56%, and median PFS of 9 and 8.6 months. Thus all these trials, including our own with over 100 patients, are highly consistent in reporting a level of efficacy that may be slightly superior to that seen with the 3-weekly taxane regimen. The only randomized comparison of 3-weekly vs weekly paclitaxel comes from the CALGB 9840 study, which was also amended to incorporate trastuzumab for all HER2-positive patients and on a randomized basis for HER2-negative patients [22]. The dose-dense schedule showed significantly superior response and time to progression, plus a distinctly favorable trend in overall survival and, as expected, no major antibody benefit in HER2-negative patients.

Two phase II studies adding carboplatin to paclitaxel and trastuzumab suggested even greater efficacy with response rates >80% and median PFS markedly exceeding 1 year [23,24]. However, these results were achieved in first-line patients only and must be weighed against substantial grade 3 to 4 myelosuppression. Weekly docetaxel + trastuzumab also appears a feasible combination but has been investigated in very few patients [25].

As for safety, the randomized CALGB trial [22] suggested that weekly, uninterrupted paclitaxel caused more neuropathy and less hematotoxicity than the conventional schedule, reflecting the clinical challenge of balancing toxicity and efficacy in the optimization of palliative treatment. However, in contrast to the other phase II/III studies of weekly trastuzumab/paclitaxel [18,20-22] which maintained the cytotoxic component until progression or toxicity in most patients, we preferred to introduce a two weeks break and, more importantly, to limit the duration of chemotherapy so that it eventually lasted for less than one third the median total duration of trastuzumab treatment. As a consequence, we had to terminate treatment because of toxicity in only 6% of patients, while the total median duration of trastuzumab treatment (42 weeks) was distinctly longer than in the other phase II trials that reported median therapy periods of less than half a year. This was consistent with the low rate of severe sensory neuropathy (5%), contrasting with the approximate 25% reported by Seidman et al. [20,22]. Despite the anthracycline pretreatment in all our patients, cardiotoxicity was rare, with clinical symptoms reported in two cases only. This compares favorably with the 11% grade 3–4 myocardial dysfunction found in the pivotal study with 3-weekly paclitaxel [6]. The other phase II trials of weekly paclitaxel reported rates of 2–8% but prior anthracycline was not a mandatory selection criterion in the two larger studies [18,20].

We consider our schedule combining two 6-week courses of paclitaxel with concurrent and continued trastuzumab to be a highly effective option feasible in the vast majority of patients despite anthracycline pretreatment. The finding in a recent meta-analysis that continuation of first-line chemotherapy until disease progression has at best a marginal effect on overall survival in most patients [26] only bolsters the rationale for our de-escalated approach.

## Competing interests

The authors declare that they have no competing interests.

## Authors’ contributions

All authors have made substantial contributions to the conception of the trial and the acquisition of data; they participated in the critical revision process of the manuscript and approved the final version. MJ was the principal study coordinator; he designed the trial and its protocol, and was involved in manuscript writing. AH was involved in the protocol development and manuscript writing, managed the database and was responsible for the biostatistical planning and analysis. All authors read and approved the final manuscript.

## Pre-publication history

The pre-publication history for this paper can be accessed here:

http://www.biomedcentral.com/1471-2407/12/165/prepub
